# 2173. Activity of Ceftobiprole Against *Enterococcus faecalis* Clinical Isolates From the United States (2016–2020), Including Those From Difficult-to-Treat Infections

**DOI:** 10.1093/ofid/ofad500.1795

**Published:** 2023-11-27

**Authors:** Rodrigo E Mendes, Leonard R Duncan, Helio S Sader, Jennifer Smart, Mark E Jones, Mariana Castanheira

**Affiliations:** JMI Laboratories, North Liberty, Iowa; JMI Laboratories, North Liberty, Iowa; JMI Laboratories, North Liberty, Iowa; Basilea Pharmaceutica International Ltd, Allschwil, Basel-Landschaft, Switzerland; Basilea Pharmaceutica International Ltd., Allschwil, Switzerland, Allschwil,, Basel-Landschaft, Switzerland; JMI Laboratories, North Liberty, Iowa

## Abstract

**Background:**

Ceftobiprole (BPR) is an advanced-generation cephalosporin that has *in vitro* and *in vivo* activity against clinically important Gram-positive organisms, including MRSA and Gram-negative organisms. While BPR does not have clinically relevant activity against *Enterococcus faecium*, BPR is active against *E. faecalis*, an opportunistic bacterial pathogen of increasing clinical relevance and a significant cause of infective endocarditis. This study evaluated the activity of BPR and comparator agents against *E. faecalis* isolated from patients hospitalized in US medical centers.

**Figure:**
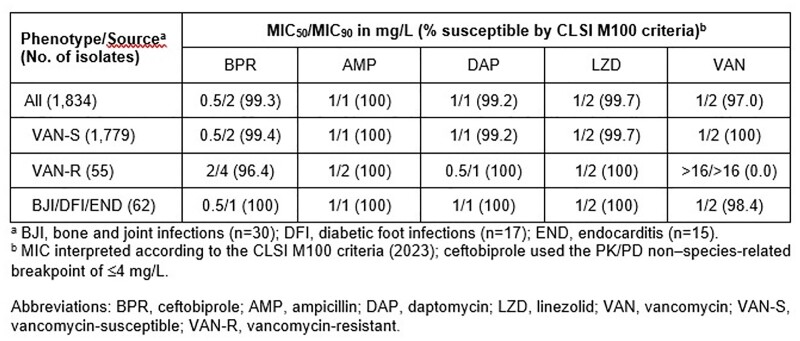

**Methods:**

A total of 1,834 *E. faecalis* isolated from 34 US medical centers (2016–2020) were included. Isolates collected were responsible for bloodstream infections (40.8%), urinary tract infections (23.4%), skin and skin structure infections (22.0%), and other infection types (13.9%), including bone and joint infections (BJI; 1.6%), diabetic foot infections (DFI; 0.9%), and endocarditis (END; 0.8%). Isolates were tested for susceptibility using the CLSI broth microdilution method and MIC interpretations followed CLSI criteria. For BPR, the PK/PD non–species-related breakpoint of ≤4 mg/L was used.

**Results:**

BPR inhibited 99.3% of all *E. faecalis* at ≤4 mg/L, whereas the comparator agents, ampicillin (AMP), daptomycin (DAP), linezolid (LZD), and vancomycin (VAN), inhibited between 97.0% to 100% of all isolates at their respective breakpoints. A total of 3% of all *E. faecalis* isolates were VAN resistant. BPR had similar MIC_90_ results of 2 mg/L and 4 mg/L when tested against VAN-susceptible and -resistant isolates, respectively. AMP, DAP, and LZD were also active against VAN-susceptible and -resistant *E. faecalis.* All (100%) isolates causing difficult-to-treat infections, such as bone/joint and diabetic foot infections and infective endocarditis, were inhibited by BPR at ≤4 mg/L in addition to AMP, DAP, and LZD at their respective breakpoints.

**Conclusion:**

These data suggest that BPR represents a potential option for empirical and guided treatment of infections caused by *E. faecalis* in US hospitals, including isolates causing difficult-to-treat infections.

**Disclosures:**

**Rodrigo E. Mendes, PhD**, AbbVie: Grant/Research Support|Basilea: Grant/Research Support|Cipla: Grant/Research Support|Entasis: Grant/Research Support|GSK: Grant/Research Support|Paratek: Grant/Research Support|Pfizer: Grant/Research Support|Shionogi: Grant/Research Support **Leonard R. Duncan, PhD**, AbbVie: Grant/Research Support|Basilea: Grant/Research Support|CorMedix: Grant/Research Support|Melinta: Grant/Research Support|Pfizer: Grant/Research Support **Helio S. Sader, MD, PhD, FIDSA**, AbbVie: Grant/Research Support|Basilea: Grant/Research Support|Cipla: Grant/Research Support|Paratek: Grant/Research Support|Pfizer: Grant/Research Support|Shionogi: Grant/Research Support **Jennifer Smart, PhD**, Basilea Pharmaceutica International Ltd, Allschwil, Switzerland: Stocks/Bonds **Mark E. Jones, PhD**, Astellas Pharma Global Development, Inc: Support for the present publication|Basilea Pharmaceutica International Ltd: Employee of Basilea Pharmaceutica International Ltd|Basilea Pharmaceutica International Ltd: Stocks/Bonds **Mariana Castanheira, PhD**, AbbVie: Grant/Research Support|Basilea: Grant/Research Support|bioMerieux: Grant/Research Support|Cipla: Grant/Research Support|CorMedix: Grant/Research Support|Entasis: Grant/Research Support|Melinta: Grant/Research Support|Paratek: Grant/Research Support|Pfizer: Grant/Research Support|Shionogi: Grant/Research Support

